# Dr. Thomas on Chronic Affections of the Digestive Organs

**Published:** 1821-09-01

**Authors:** 


### VIII.

I. *Practical Observations on Chronic Affections of the Digestive Organs, and on Bilious and Nervous Disorders; being an Attempt to combine with English Practice, some useful Methods of Cure employed on the Continent. Also, Remarks on Warm Mineral Waters in general, and on the Use and Abuse of the Cheltenham Waters.* By JOHN THOMAS, M. D. many years Resident Physician at Toulouse, now practising at Cheltenham. Octavo, pp. 168. Cheltenham, 1820.

II. *Dictionnaire des Sciences Medicales,* Vol. V. article "*Clysma.*" Par M. BARBIER.

III. *Dictionnaire des Sciences Medicales,* Vol. XLIX, article "*Sangsue.*" Par M. MERAT, M. D.

THE attempt to introduce improvements from foreign countries into our own, is, at least, meritorious; and how far Dr. Thomas or ourselves may be successful in this way, time must decide. In the mean while it is our duty first to lay before the public an outline of our author's labours.

Dr. Thomas informs us that he has had extensive practice, for many years, in a southern climate, and that his experience there has verified the pathology and therapeutics of Dr. Hamilton and Mr. Abernethy, whose writings he greatly eulogizes.

"In the slighter pathology or mere functional disorders of the digestive or chylopoiëtic organs, the only addition I have been under the necessity of making to the use of the blue pill, calomel, or some drastic purgatives in small doses, was that of clysmas, and warm baths,—but in chronic inflammations, or congestions of the different cavities, particularly that of the abdomen, I have found much advantage in depleting those vessels that contribute to feed the *vena portarum*, as well as other veins in a state of congestion in the tissues or parenchyma of the abdominal viscera." 9.

The first case brought forward by Dr. Thomas is in illustration of the effect of leeches applied to the anus, with blue pill and drastic purgative medicines internally.

Mr. D———, æt. 30, of Toulouse, naturally of delicate constitution, had been suffering under ascites and anasarca for upwards of two months, attended by a respectable physician of the above city, who considered the disease a slow conversion of external rheumatism upon some internal organ. Our author, on examination, could find nothing but a fluctuation in the abdomen, the parietes were so stretched. There was an emphysema between the third and fourth, and between the fourth and fifth ribs, on the right side of the thorax. The French physicians, from this circumstance, concluded that the disease was in the right lobe of the lungs. On the 8th of September, 1810, when our author first saw the patient, his tongue was white and dry, pulse weak and irregular, skin sometimes hot, sometimes cold, urine high-coloured and scanty, bowels costive, nights sleepless, respiration difficult and laborious, strength exhausted, no appetite. His French regimen was bouillon every four hours, with a table spoonful of wine immediately upon it.

After an enema, and some draughts with the acetate of ammonia, Dr. Thomas prescribed a decoction of taraxacum and gratiola, with supertartrite of potash, to which the French apothecary added some lean of veal. This medicine produced daily some copious alvine evacuations, generally of a depraved kind. The urine became more plentiful, the pulse more regular and full;—" in short, a general amendment and an increase of strength were obviously observed to keep pace with the evacuations." The abdomen soon became so reduced that the state of the individual organ could be ascertained; and, on examination, the liver was found to be " much enlarged," and to be felt full two inches below the false ribs.

" The emphyzema on the right side of the thorax appeared no longer problematic, the diaphragm being forced into that cavity by the increased volume of the liver producing that phenomenon." 19.

We really are at a loss to know what Dr. Thomas can mean by emphysema in this case. The word, both from its Greek derivation* and common application, means an elastic swelling caused by the introduction of air into the cellular membrane. How an enlarged liver could produce this effect between the ribs is beyond our conception.

Our author now ordered eight leeches to be applied to the anus, and five grains of the quicksilver pill to be taken every night, continuing the medicated broth before described. In four days—

" The liver sensibly diminished—the stools approaching their natural colour—urine plentiful—the anasarca nearly dispersed,—no fluctuation perceptible in the abdomen, and the skin acquiring that softness inseparable with [from] health—the pulse 80 and regular. The patient expressed a desire to eat—the breast of a roasted chicken was allowed him, which he ate with great pleasure with the end of a roll and a glass of wine and water." 20.

The same remedies were persisted in, with the addition of another leeching to the anus, and a blister to the region of the liver. The patient shortly recovered, by a perseverance in the blue pill, and simple decoction of dandelion.

We suspect, from some little experience, that enlarged livers and consequent dropsical effusions, will not generally be found so tractable as in the above case. At the same time we have nothing to object to the mode of treatment pursued.

The next case detailed by Dr. Thomas, is that of a cattle drover, of irregular habits, who had been complaining for some months of various dyspeptic symptoms, which so often mark hepatic derangement; such as anorexia, acid eructations, palpitation of the heart, languor, heartburn, dejection of spirits, head-ache, emaciation, debility. The complaint had been treated as nervous, and unsuccessfully. Our author found him, on the 13th October, 1813, with the following symptoms.

"I found his skin hot and dry, his tongue much furred with a buffy or yellow tinge, his pulse small, weak, ranging from 100 to 110, no appetite, a bitter taste on the tongue, much thirst, head-ache, a pain on the top of the right shoulder, his bowels very irregular, generally costive, but sometimes passing a small quantity of alvine fluid, perfectly characteristic of those partial excretions often met with in deranged functions of the colon, the urine scanty and high-coloured; on examining the state of the abdomen, I easily discovered the liver to be preternaturally large, and its convex surface very sensible to the touch. The patient had been ill nearly eight months, and was much reduced in strength—and towards evening the feet and ancles were a little swelled. I began by ordering the application of eight leeches to the anus, and the next morning an ounce and a half of the oleum ricini was given him in a little broth; he had two copious evacuations of dark greenish matter, with scybala." 25.

Calomel and rhubarb, blue pill, leeches to the anus, blister to the hepatic region, the warm bath, decoction of dandelion with carbonate of soda, restored him to health in little more than a month.

In the next case, a man of rather irregular habits, our author found considerable enlargement of the liver, with sallow complexion, yellow conjunctiva, habitual costiveness, scanty urine, frequent, small, weak pulse, skin dry and hot, decubitus difficilis on the left side, failure of strength and appetite, sense of fulness after taking the least nourishment.

Glysters, leeches to the anus, aperients, blue pill, and diluents, until the mouth became sore.

"When his mouth became sore, I judged it necessary to discontinue the blue pill; his pulse was now reduced to 80, his stools regular, and approaching a natural colour, his urine much improved in colour and in quantity; the skin had resumed its natural feel, and had it not been for his mouth, he said he could eat a hearty dinner." 33.

In a month from the time he came under Dr. Thomas's care, he returned home pretty well. We shall not enter into the analysis of any more of the cases detailed in this volume. The foregoing will convey a very good idea of what our author's practice was, and to what point his therapeutical indications tended.

"I have dwelt, some may say too long, on the advantage of depleting the abdominal venous system by the application of leeches to the anus. I have insisted on the usefulness of clysmas. I shall conclude by recommending the combination of warm mineral baths, with the internal use of mineral waters in simple functional disorder of the digestive organs, and by pointing out the necessity of employing that combined treatment to consolidate the cure of chronic affections of any of those organs, after the oppression occasioned by the too great accumulation of blood in that organ, shall have been relieved by depleting the abdominal venous system, and other means already mentioned." 91.

Our author informs us that tartrite of antimony is a medicine much used in France, given in very minute doses: for instance, one grain in eight common wine bottles of water; a bottle to be drunk in the 24 hours, and this continued for weeks or months. This dilute proportion is found useful in incipient affections of the chest.

Dr. Thomas praises, as may be naturally supposed, the waters of Cheltenham, as alteratives and aperients, "sufficiently strong to fulfil all the indications a rational and experienced physician can require." The muriates and carbonates are esteemed the most valuable of the component parts of these waters, but their virtues, he thinks, are enhanced by the small proportion of sulphates that enter into combination with them, and prevent their irritating effects by keeping the bowels open. Our author's observations on these waters, and their chemical analysis, taken from Dr. Scudamore's work, we shall pass over, of course, in order to notice certain continental mineral springs. Most of those glanced at by our author are of temperature, varying considerably in degree. None of them, he observes, are so highly impregnated with saline principles as the Cheltenham waters. "Balaruc comes the nearest in quantity of salt held in solution." These waters are, in temperature, as high as 118°. By being prescribed in baths, at the same time that they are internally administered, they produce a purgative effect without irritating. These waters are celebrated in bilious and paralytic affections. The waters of Spa contain much less saline ingredients than those of Cheltenham, but they are highly gaseous.

"Nothing is more easy than to convert Cheltenham waters into Spa water; if to one part of Cheltenham waters at the bottom of a glass you pour two parts of soda water, so pleasant and so fashionable a beverage, you will then have Spa water in no respect inferior to the original." 135.

About four leagues from Bagnares are situated the most renowned and ancient mineral waters of the Pyrenees—Bareges, where Cæsar and Sertorius drank from the salubrious springs, and erected noble monuments.

"Bareges is situated in a valley of that name in the heart of the Pyrenean mountains, it is covered with snow more than half the year; there are only a few houses inhabited during the winter; the different tradesmen repair there in the summer, and occupy temporary huts for the space of four or five months, and the company in general is very indifferently accommodated, excepting those persons who arrive early in the season, and engage the few decent apartments to be found in the place. The country is barren, and inspires every other idea but that of comfort; yet such is the reputation of these waters, that persons of the highest rank of both sexes flock to them from all parts of Europe. I have known some highly distinguished characters from our own country who visited Bareges, many of whom derived much benefit from the use of the waters.

"No exact analysis has been made of these waters, though many have attempted it; however it is clearly ascertained that they contain sulphuretted hydrogen gas, muriate of soda, carbonate of soda, an absorbent earth, alumine, and bitumen, but all in a very small quantity; they are of different degrees of temperature, some hot, some warm, and some temperate; they vary from about 90 to nearly 140 of Farenheit." 139.

Although containing less salts than the waters of Cheltenham, yet the bathing and drinking combined produce effects sufficiently purgative, and other means of keeping the bowels open are seldom necessary.

"The waters and baths of Bareges are perfectly imitated at Tivoli, in Paris, and from several conversations that I have had with some eminent physicians in that capital, they appeared quite satisfied with their effect. If then they have been brought to perfection at Paris, there is much reason to presume that they may be effected at Cheltenham, where the principal ingredient is already at hand, the Cheltenham water; the caloric, sulphuretted hydrogen gas, &c. could be easily added, and English patients might be indulged with Bareges, or any other baths, without foregoing the comforts of their own country." 140.

Dr. Thomas thinks that the warm bath is in far too little use in this country, and that when employed, the immersion is not long enough continued. "What effect can be expected from a warm bath of ten or at most fifteen minutes, which is the general practice in England?"

"Upon the Continent, where so much good is produced by this powerful remedy, no one thinks of recommending a patient to stay less than an hour in the warm bath; and at Ussat, where so many cures have been effected by means of the bath, and by such means *only*, I have known many weakly delicate patients take two baths of one hour each every day for three weeks without intermission; and I have no doubt in my mind, and the same conviction pervades medical men in general in France, that it is owing to this manner of taking the warm mineral baths, so much good is effected by that remedy; and by parity of reasoning, I may say that little good is obtained from them in this country, because there is not sufficient time allowed for bathing." 151.

We must now conclude our analysis. The chief object,—and we might say, the chief merit, of the work before us, consists in recommending strongly to the practitioners of this country the auxiliaries of anal leeching, enemata, and the warm bath, as practised on the Continent. Our readers are well aware that, in the different series of this Journal, we have repeatedly drawn their attention to these subjects. In doing so now again we may be accused of tautology; but we shall here, once for all, state it as our firm conviction, that, in medicine, as in religion, the great and fundamental truths must be not only brought forward, but *reiterated* from time to time, so as to be kept constantly before the eyes of the young who are backward enough in learning them, and the old who are forward enough in forgetting them.

Under this conviction we shall here introduce some observations drawn from our own experience, reflections, and researches, on subjects which are far too little studied or attended to in this country.

*Enemata.* The rectum and colon to which glysters are applied, present a very extensive surface, endued with no inconsiderable share of sensibility, being plentifully furnished with nerves, blood-vessels, absorbents, and exhalents. In this respect, it is true, that their surface falls far short of the stomach and small intestines, where the sensibility is exquisite, and the play of sympathetic associations most extensive. Yet the portion of bowel concerned in glysters is lined with a membrane that has considerable sympathetic connexion with the other great organs of the body, and may therefore be made the medium of much medicinal agency on the constitution. Every one knows that where deglutition has been impeded, the absorption from this intestine alone has long sustained the vital actions, though absorption is probably one of its weakest powers. In general the quantum of active remedies injected into the colon should be double or treble that introduced by the stomach.

The common effect of all kinds of enemata are the evacuations of the lower bowels. Even water injected by the anus excites the action of the great intestines to an expulsion of their contents. But, when medicinal agents are added to this simple menstruum, another, or ulterior, effect is produced besides the evacuation of the bowel. Suppose, for instance, an enema, containing infusion of senna, rhubarb, jalap, or colocynth, or solutions of the neutral salts, be thrown up, and allowed to remain for some time in the colon. An excitement, more or less powerful, is produced throughout the mucous membrane of the gut. The vital properties of this membrane are developed—the blood flows in greater quantity, and with greater force into the capillaries of its texture, which becomes swelled, reddened, hotter, and more sensible—in short, a determination of blood and nervous excitement is made, pro tempore, to this portion of the primæ viæ, which becomes a kind of centre of vital action for the time. Next comes on some degree of pain, and colicky contractions, followed, more or less quickly, not only by a detrusion of the contents of the small intestines into the large, but of the contents of these last also, together with the additional secretions poured out from the mucous membrane of the bowels during this artificial excitation.

This process may be very advantageously employed by the physician, in remedying or mitigating many troublesome affections. In constipation, and all the numerous ills thereon dependent, an excellent auxiliary, if not a principal remedy, is to be found in the regular use of aperient lavemens. In spasmodic dyspnœa, in head-aches, in gastrodynia, and various other cases, the fluxionary movement which is thus determined to the lower portion of the alimentary canal, becomes highly beneficial. In a case of violent colic with vomiting, M. Barbier saw instant relief produced by throwing up an injection containing the infusion of a drachm of colocynth. To females who have weaned their children, gently purgative injections are of the greatest service in preventing the ill effects arising from the suppression of the lacteal secretion. Accoucheurs know well the power of stimulating injections, where the uterus is torpid, in recalling its energies and renewing its contractile efforts.

Acrid purgative enemata have always been found useful in apoplexy, paralysis, and colica pictonum. It need hardly be said that this class of enemata are detrimental where any irritation or inflammation exists in the mucous membrane of the lower bowels in particular.

*Tonic and Astringent Lavemens* have been often employed, especially on the Continent, in chronic dysenteries and diarrhœas, in passive uterine discharges, incontinence of urine, chronic blennorhagies, &c. The cinchona is often successfully administered in this way, when it cannot be taken by the mouth.

What our continental brethren term "*exciting glysters*," are generally composed of infusions of wormwood, chamomile, horehound, sage, rosemary, baum, wild valerian, anise seed, fennel, juniper berries, turpentine, assafœtida, &c. &c. These stimulating and aromatic substances first produce an excitation of the intestinal mucous membrane, which is soon communicated to the whole nervous and vascular systems, producing an increased activity of the secretions and excretions. These agents, therefore, might be turned to much use, were the prejudices against the process obliterated, as they ultimately must be. Hoffman (De Clysterum usu Medico) recommends the above class of lavemens in many chronic diseases accompanied with debility, flabbiness of the muscles, paleness of the skin, and general want of energy in the system. But it is in colicky affections, attended with distressing flatulencies, atony of the intestines, and fœcal accumulations, that M. Barbier particularly recommends these stimulating and aromatic injections, to which may be added cathartic ingredients occasionally, as solutions of Epsom salts, aloes, or infusions of senna. We have ourselves seen great advantage from injections of this description, in the troublesome complaints abovementioned, to which sedentary females are peculiarly prone. This class of lavemens ought to be prohibited, of course, in all cases where there is febrile heat, inflammatory pain, or any increased excitement of the system.

More diffusible stimuli than the above, to wit, port wine, and even alcohol, have been used in lavemens. It is a curious fact that inebriety more quickly results from vinous or spirituous glysters than from analogous potations taken into the stomach.

*Narcotic and Anodyne Glysters* are prepared from poppy heads, opium, hyoscyamus, &c. These exert a powerful influence not only on the great intestines, but on the stomach and the whole system. In this way they allay pain without so much deranging the stomach as when they are taken by the mouth. In the pains attending nephritic and calculous complaints, we have a valuable resource in anodyne lavemens internally, and opiate fomentations to the region of the bladder, perineum, and loins. In these cases from 50 to 100 drops of laudanum in three or four ounces of starch, should be thrown up the rectum, the patient keeping quiet in bed for some time afterwards, to prevent its coming away. At the same time, a decoction of poppy-heads, to which an ounce of laudanum is added, may be applied to the pelvic region by means of flannels, in the way of a fomentation. The various cases to which anodyne injections are applicable, will easily suggest themselves to the intelligent practitioner.

What are termed, on the Continent, *laxative and emollient lavemens* are those prepared from laxative, mucilaginous, farinaceous, oily, and gelatinous substances, used by themselves, or as vehicles for other medicinal agents, as opiates, cathartics, stimulants, &c. The substances most commonly employed for the preparation of this class of injections, on the Continent, are manna, cassia fistularis, sweet oil, leaves and roots of marsh mallows, violets, linseed, barley, starch, milk, weak broth, hartshorn jelly, &c. These vehicles are often very useful where stronger medicinals are combined, the irritating effects of the latter being thereby counteracted. But the habit of using injections of this class induces an atonic state of the bowels and ultimately of the whole system; hence they are improper in relaxed fibre, chronic debility, and other states of asthenia. They are, on this account, however, of great service in the opposite kinds of maladies, namely, those attended with febrile movements, and tense fibre, as in fevers of excitement, chronic phlegmasiæ of the serous membranes, or of the parenchymatous structure of organs, rheumatism, and many spasmodic affections.

*Cold Lavemens* are of great service in many complaints, particularly in uterine hæmorrhage and painful hæmorrhoids. In the latter complaint particularly, we know, from some experience, that lavemens of *cold water*, together with exercise, on horse and foot, and regulation of the bowels by small quantities of the blue pill with supertartrite of potash, sulphur, and honey, are the most effective items in the methodus medendi. All acknowledge the importance of assafœtida, turpentine, and other injections in cases of worms. A few observations on the mode of using injections shall terminate this part of the subject. We have found no instrument so good as the common syringe with the crooked tube, so as to be used by the patient himself. In cases of common torpor of the bowels, either as a substitute or auxiliary to internal medicines, a pint of warm water, in which is dissolved a little yellow soap, will generally be sufficient to bring away a stool. We do not think it necessary to keep it in more than two or three minutes, if so long. The great object is, by the mechanical distention of the rectum and colon, to excite the action of their contractile fibres, while the current of the fluid, passing off, carries with it whatever fæces may be lodging in the lower portion of the canal. If the fluid is allowed to remain longer than a few minutes, the *conatus ejiciendi* generally goes off, and the water is absorbed, without inducing a motion. But where it is necessary to combine salts, or other aperient medicine with the water, then we consider it best to take the enema while lying on the bed, and to keep it in half an hour or more, in order that the medicines may produce their effect on the mucous membrane of the bowels, and increase their secretions, before passing off.

*Leeching*. Before closing this article, we shall introduce a few observations relative to local blood-letting by means of leeches. These useful animals were familiar to the ancients, and from their avidity for blood, were denominated sanguisugæ. Pliny informs us that elephants, who happened to swallow them, were cruelly tormented in consequence, and the human species has not escaped their ravages in this way, as has been proved on several occasions. We shall pass over the natural history of the officinal leech. It is difficult to say on what these creatures live, except it is the water itself; for their sanguisuction appears an act of unparalleled gluttony, which they carry to such an excess as destroys them, unless they are forcibly disgorged. It is curious that if there be too many of them in a vessel, in proportion to the quantity of water, the stronger will destroy the weaker, and devour them for sustenance. The more water there is about leeches the better they are preserved. One hundred leeches should have at least three quarts of water. It should be changed twice a week in summer, and once a week in winter. In very hot weather it must be changed every two days. Leeches ought also to be washed occasionally, to cleanse them of the mucous and excrementitious substances which adhere to them. By putting them on a sieve, fresh water may be poured over them till they are clean; and if this will not do, they must be cleaned individually with the hand. The vessels in which they are kept should be very carefully cleansed, and exposed for some time to the pure air to dissipate any disagreeable odour which is prejudicial to the leeches. Dead individuals ought to be immediately removed, otherwise they corrupt the water. The vessels in which leeches are kept should not be exposed in windows to the rays of the sun. The cooler they are kept the better, provided it does not approach the freezing point. When changed, water of the same temperature is to be used. Nothing is so destructive to leeches as vicissitudes of temperature in the fluid they inhabit. It is a curious fact, that these animals have been frozen for a month together, and gradually thawed, with preservation of life. When this accident happens, therefore, they are not to be thrown away.

In order that leeches may bite readily and suck well, they should be made to *fast* for a few hours before they are applied—that is, they should be taken out of the water, and put into a cup, tied over with linen, for four or five hours in winter, and three or four in summer. They are then to be put into a dry and fine napkin, and gently rubbed, which irritates them, and makes them bite keener. If it be winter, they are the better to be breathed upon, for a minute or two, before application, the warmth of the breath making them more lively; or the vessel in which they are kept may be placed near a fire for a short time, so as to raise the temperature of the water a little, a great degree of cold rendering them torpid.

The number of leeches to be applied must vary according to the exigency of the case, and the age of the subject. To an infant of very tender age, it is not quite safe to apply more than one, two, or three. As years advance the number increases to 24 or 30. We are rarely authorized to exceed this at one application. Some of the Broussaian school, however, have applied as many as 250 in 24 hours to one patient. *Several hundred thousand* leeches were employed in the Parisian hospitals alone, in the year 1819.

There is no part of the body, almost, to which leeches may not be applied. To the anus and ostium vaginæ they are very seldom applied in this country, but very often on the Continent. The best rule is, to apply the leeches as nearly as possible to the seat of inflammation or pain. A

part to which leeches are to be applied, should be carefully deprived of hair, which greatly obstructs their operations. The part also should be wet with a little milk, or sugar and water, which facilitates the biting, though it is much neglected in this country, where the management of leeching is not nearly so well understood as in some other countries. When they ramble about much, and are not inclined to bite, they must be put into a wine glass, and the vessel reversed on the part. By this means they will generally be made to take in a little time. When they are fixed, they should be kept perfectly quiet, and covered with a fine napkin, over which a piece of flannel is to be thrown, in order to defend the patient from cold, especially if the application be to the chest or abdomen for internal inflammation. Leeches generally require the space of an hour to fill themselves completely, when they fall off, and usually forfeit their existence as the price of their gluttony. Some of these animals go to sleep when they are full, and yet keep hold of the part. When this is the case, the edges of their lips are to be raised with the point of a pin, and they then fall off immediately. Some people cut off the tails of leeches, and allow the blood to run off there. This is a useless proceeding; for, in three cases out of four the leech lets go its hold when cut, and if it does not, the bite would bleed just as much as the wounded leech.

If by causing the leech to vomit up its repast by means of vinegar or salt, its life can be preserved, so much the better —in this country where leeches are scarce and dear. On the Continent they seldom take this trouble. It is rare that we are anxious to stop the hæmorrhage from leech-bites. On the contrary, we are generally anxious to promote it, which may be done by sponging the part frequently with warm water. The sponge or towel should not be *rubbed* on the bites, as the lips of the wounds become everted and painful by this means: In the intervals of sponging, the bites should be covered with a soft napkin till it becomes soaked with blood, when it is to be removed, and after sponging replaced by another. When leeches are put to the anus, pudendum muliebre, or other part where warm water can be applied, then the hip-bath or semicupium is to be recommended.

Among the accidents attending leeches is the misfortune of swallowing them, as happened to some of the French soldiers; for example, in Egypt. If they adhere to the fauces, or where they are within reach, they should be seized by the forceps and extracted. If they are beyond reach, salt water, or vinegar and water should be swallowed, and after some time, an emetic administered. If they insinuate themselves into the rectum or vagina, then injections of the above description should be thrown up.

Sometimes an excessive pain and irritation accompany, but rarely succeed the leech-bites, owing to idiosyncrasy of constitution. Nothing can be done in such cases but apply warm anodyne applications, if the pain continues. After a considerable bleeding by leeches, especially if the patient does not keep quiet and horizontal for some time, there is occasionally faintness, as after venesection. It only requires quietude, some light restorative, and the recumbent position.

It is a curious fact, which is worth attending to, that if food be taken soon after a leeching to any extent, and before the equilibrium of the blood is, as it were, reposised, a well-marked indigestion ensues, manifested by paleness of the face, weakness, anxiety, vertigo, sickness at stomach, and sometimes a diarrhœa;—symptoms which go off in 24 hours, if not imprudently kept up by too much food.

Many instances of obstinate, and a few of fatal, hæmorrhage have occurred after leeching. When the ordinary means of restraining the flow of blood fail and danger be apprehended, especially in the case of infants, the actual cautery should be had recourse to—a hot knitting-needle or probe will answer the purpose very well.

It appears to us that, on the Continent, especially in France, local bleeding, by means of leeches, is too much trusted to, where general blood-letting ought to precede or accompany it; while in England, phlebotomy is too exclusively employed, without the assistance of leeching. We are of opinion that in all inflammatory affections of the cranical, thoracic, abdominal, or pelvic viscera, general blood-letting should first break the force of the disease, while leeching or cupping should be called in as an auxiliary, and to save profuse effusion of blood from the general system. In all purely local inflammations, excepting of the skin itself, as in some kinds of erysipelas, leeching is the principal measure. Some kinds of ocular phlogosis, however, require general bleeding, to save the structure of so delicate an organ. Leeching principally affects the capillary system of vessels, and is not of sufficiently quick operation where a vital viscus or important internal structure is the seat of inflammation. Yet in peritoneal and pleuritic phlogosis leeches come so near the inflamed part, that they are of essential service as auxiliaries to general blood-letting and other depletory measures.

In all those cases of local plethora or congestion short of inflammation, so commonly attendant on organic affections, especially of the heart or large vessels, leeching stands pre-eminently useful. These local congestions are mostly conspicuous about the head, threatening or producing apoplexy; and leeching becomes an important preventive check to this formidable event. We need not dilate on the paramount efficacy of leeching in those local inflammations of the sub-cutaneous tissues, and of the joints, whether arising from constitutional diathesis, accidental violence, or surgical operations. These are sufficiently appreciated in this country by every surgeon.

Local bleeding, by means of leeches or scarificators, is peculiarly useful in persons of delicate constitutions, in infants, females, and old people, where general blood-letting may not be deemed necessary.

In producing what is termed a *derivation* of blood from one part to another, there is something in leeching which seems to unite the advantages of general blood-letting and blistering, to a certain extent. The blood drawn by a considerable number of leeches affects the whole vascular system, while the irritation of the bites keeps up a determination to the part itself.

Here we rest for the present. To those who may consider that we have been too minute on such a trifling topic as that of leeching, we offer the following sentiment of Merat:—" Ces precautions que nous avons detaillées avec soin ne paraîtront minutieuses qu'a ceux qui ne savent point que tout est important en médicine lors qu'il s'agit de soulager."

